# Supplementary material for: Synthesis of New Liquid-Crystalline Compounds Based on Terminal Benzyloxy Group: Characterization, DFT and Mesomorphic Properties
Source: Molecules. 2023 Apr 28;28(9):3804. doi: 10.3390/molecules28093804 (PMC10179744; doi:10.3390/molecules28093804)
Supplement: Supplementary file 1 [file molecules-28-03804-s001.zip › molecules-2352622-supplementary.pdf]

Supplementary Materials

Synthesis of New Liquid-Crystalline Compounds Based on Terminal Benzyloxy Group: Characterization, DFT and Mesomorphic Properties

Alaa Z. Omar [1], Mohammed L. Alazmi [1], Mai S. Alsubaie [1], Ezzat A. Hamed [1,*], Hoda A. Ahmed [2,*] and Mohamed A. El-Atawy [1,3]

Instruments and Apparatus

Melting points were determined by MEL-TEMP II melting point apparatus in open glass capillaries. The IR spectra were recorded as potassium bromide (KBr) discs on a Perkin-Elemer FT-IR (Fourier-Transform Infrared Spectroscopy), Faculty of Science, Alexandria University. The NMR spectra were carried out at ambient temperature (~25 °C) on a (JEOL) 400 MHz spectrophotometer using tetramethylsilane (TMS) as an internal standard, NMR Unit, Faculty of Pharmacy, Mansoura University. Elemental analyses were analyzed at the Regional Center for Mycology and Biotechnology, Al-Azhar University, Cairo, Egypt.

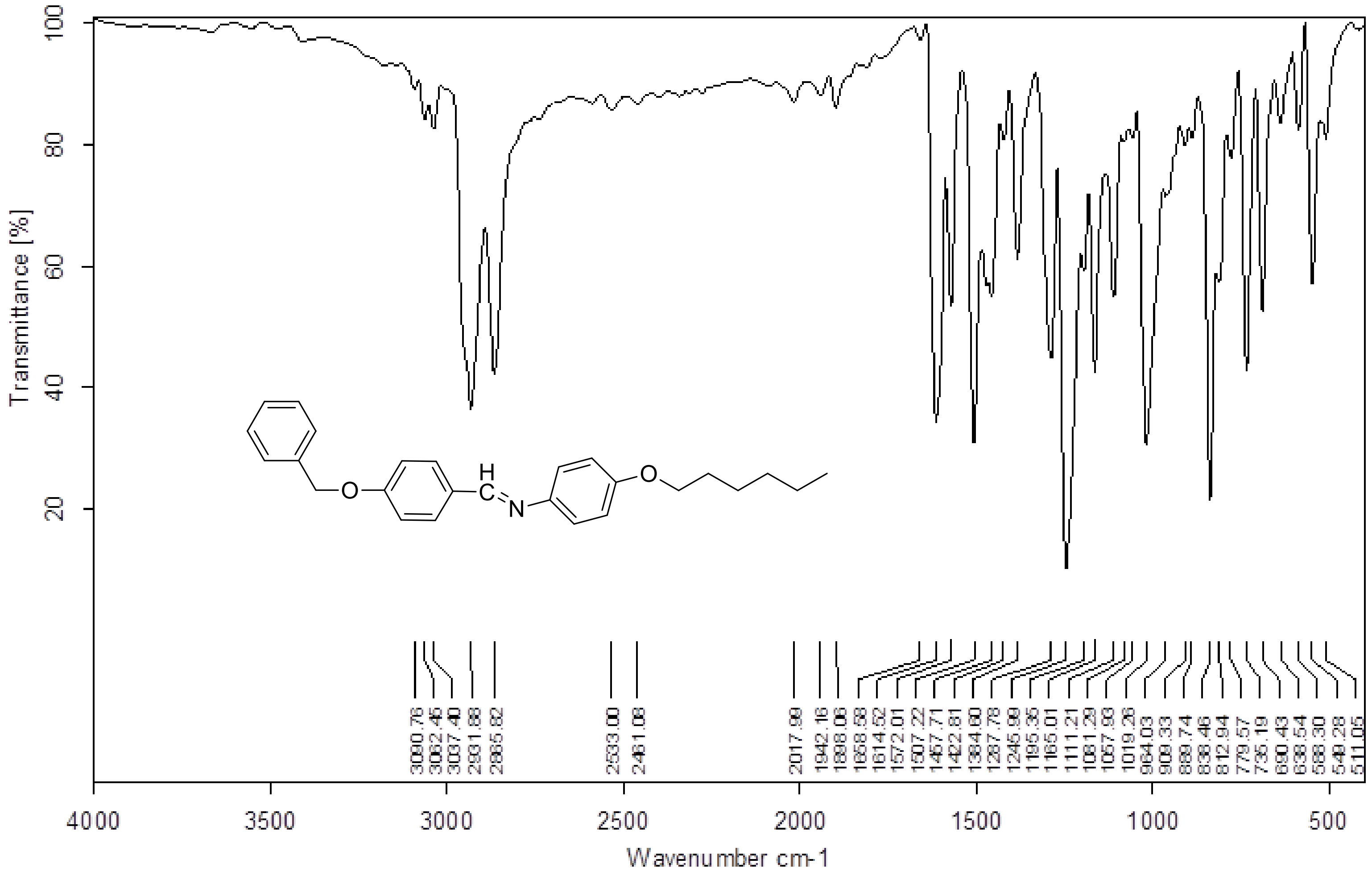

Figure S1. IR (KBr) spectrum of (*E*)-4-(hexyloxy)-*N*-(4-(benzyloxy)benzylidene)aniline **I₆**

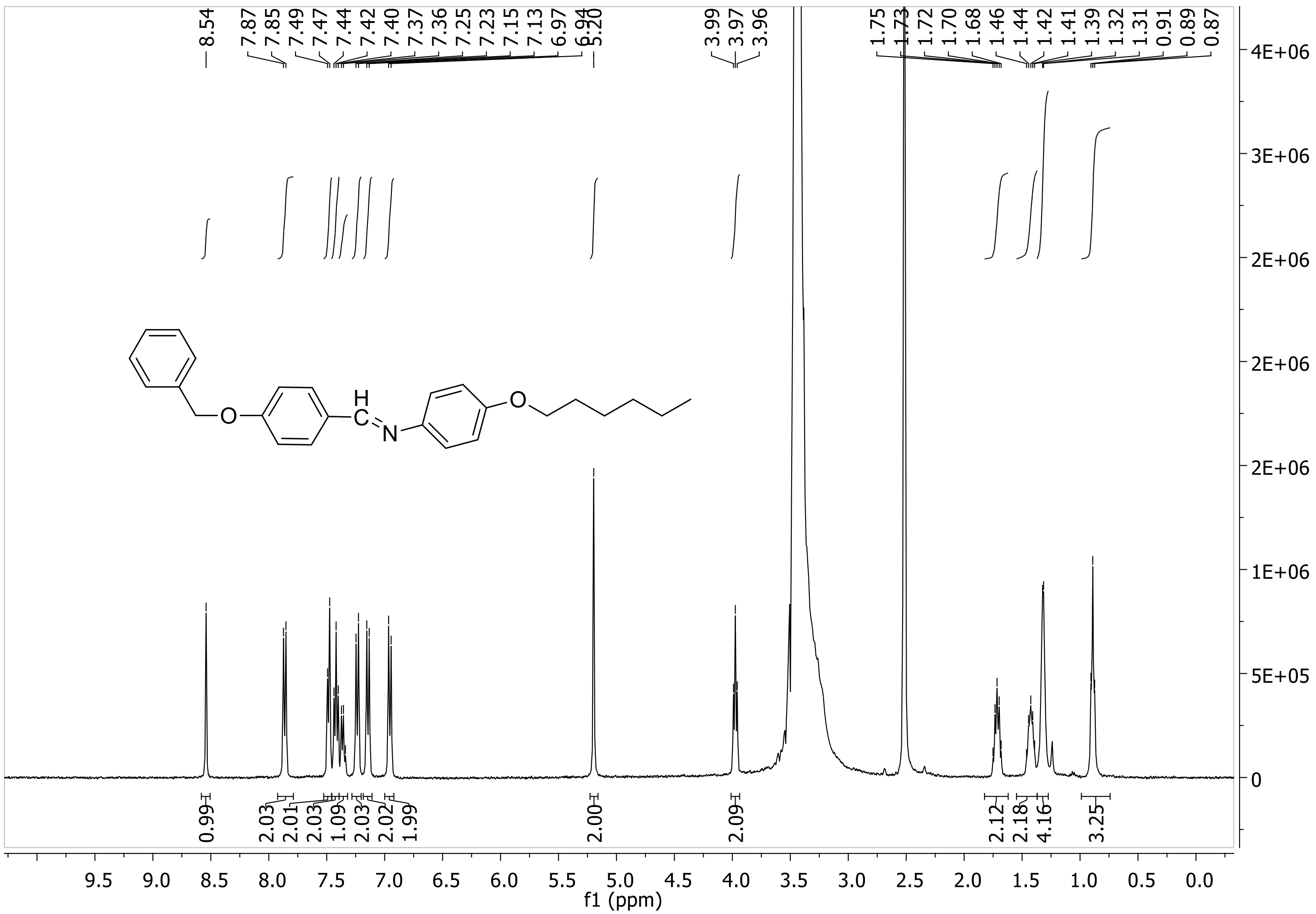

Figure S2. ^{1}H NMR (DMSO-*d6*) spectrum of (*E*)-4-(hexyloxy)-*N*-(4-(benzyloxy)benzylidene)aniline $\mathbf{I_6}$

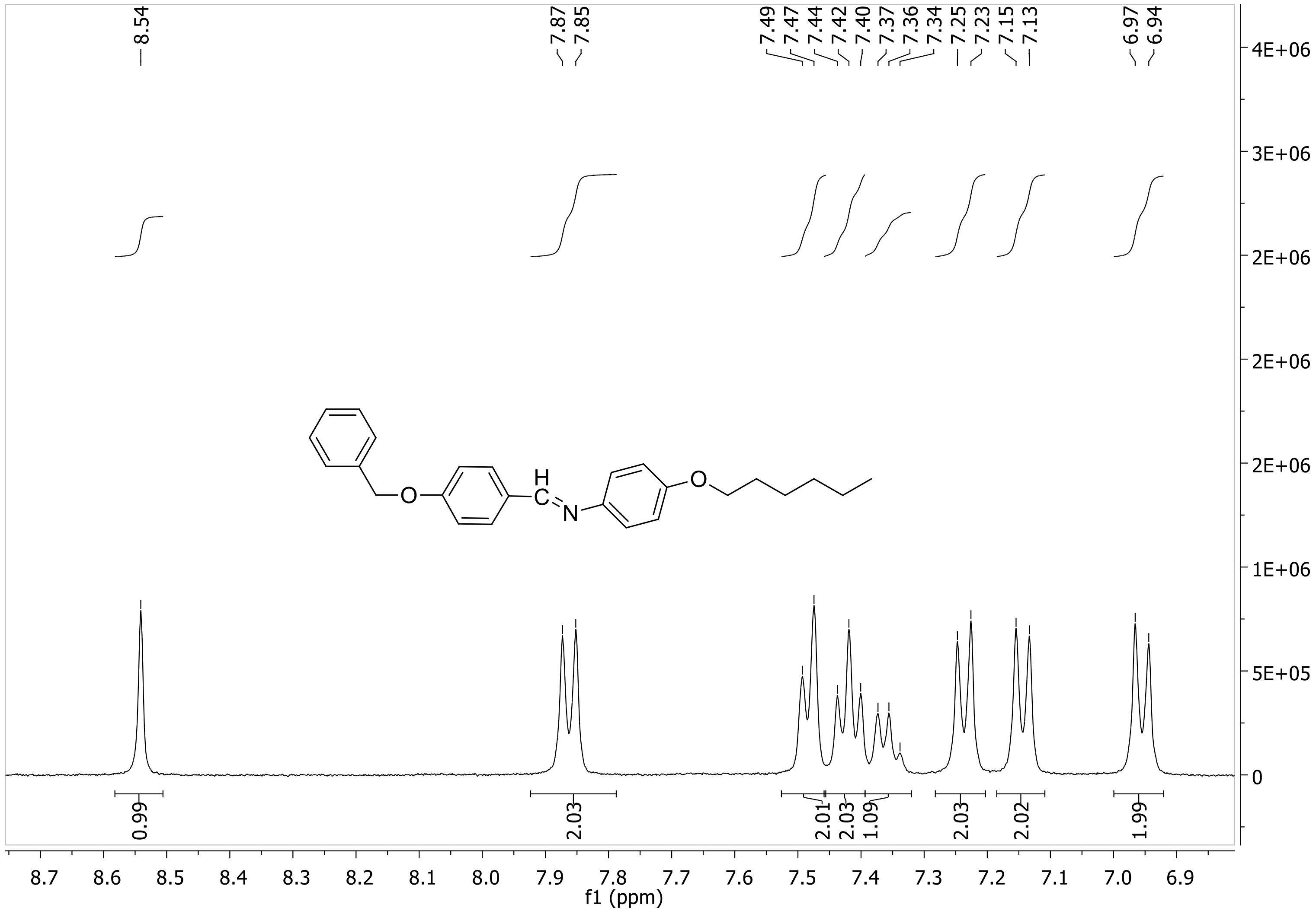

Figure S3. ^{1}H NMR (DMSO-*d6*) spectrum of (*E*)-4-(hexyloxy)-*N*-(4-(benzyloxy)benzylidene)aniline $\mathbf{I_6}$

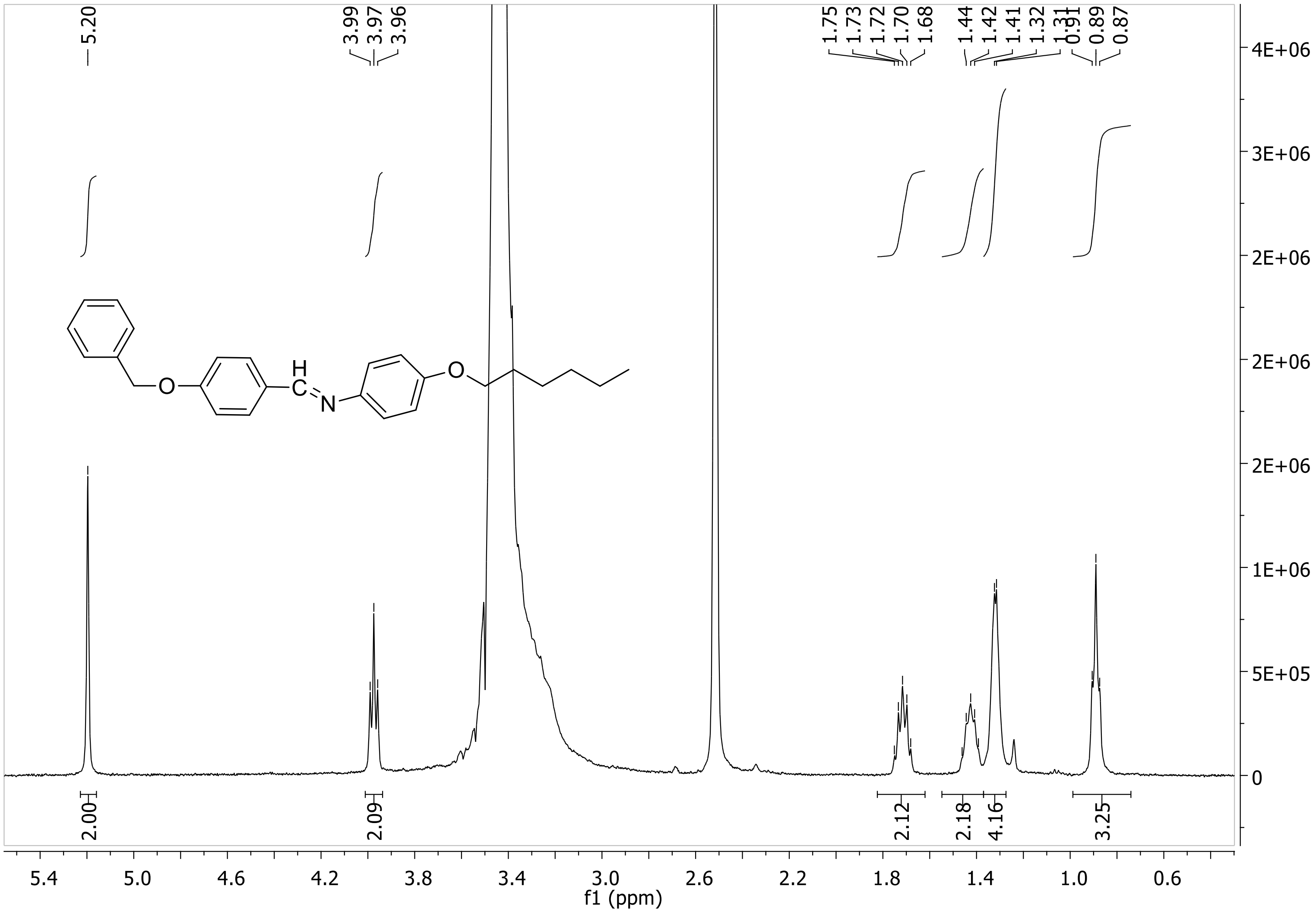

Figure S4. ^{1}H NMR (DMSO-*d6*) spectrum of (*E*)-4-(hexyloxy)-*N*-(4-(benzyloxy)benzylidene)aniline $\mathbf{I_6}$

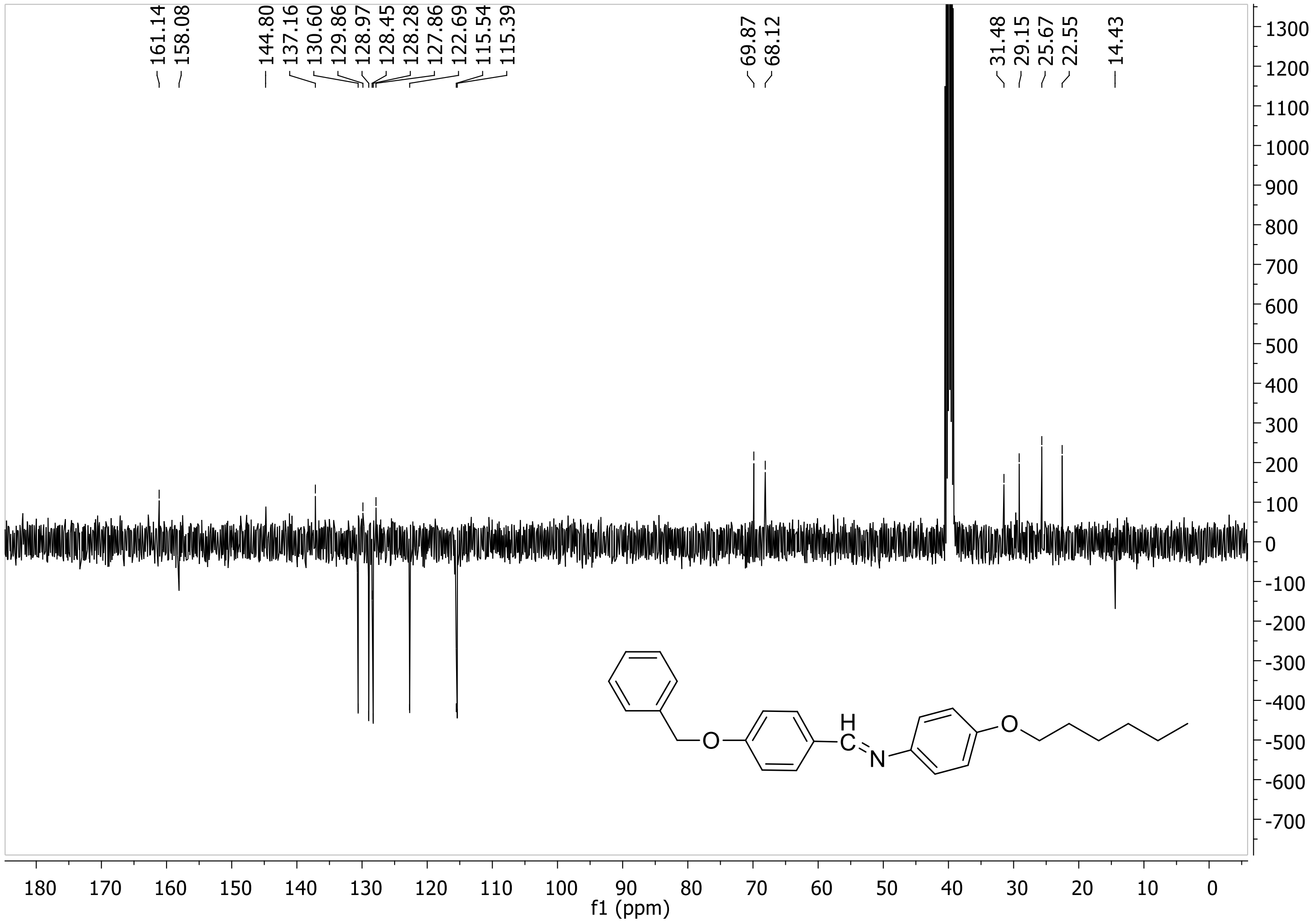

Figure S5. ^{13}C NMR (DMSO-*d6*) of (*E*)-4-(hexyloxy)-*N*-(4-(benzyloxy)benzylidene)aniline **I$_6$**

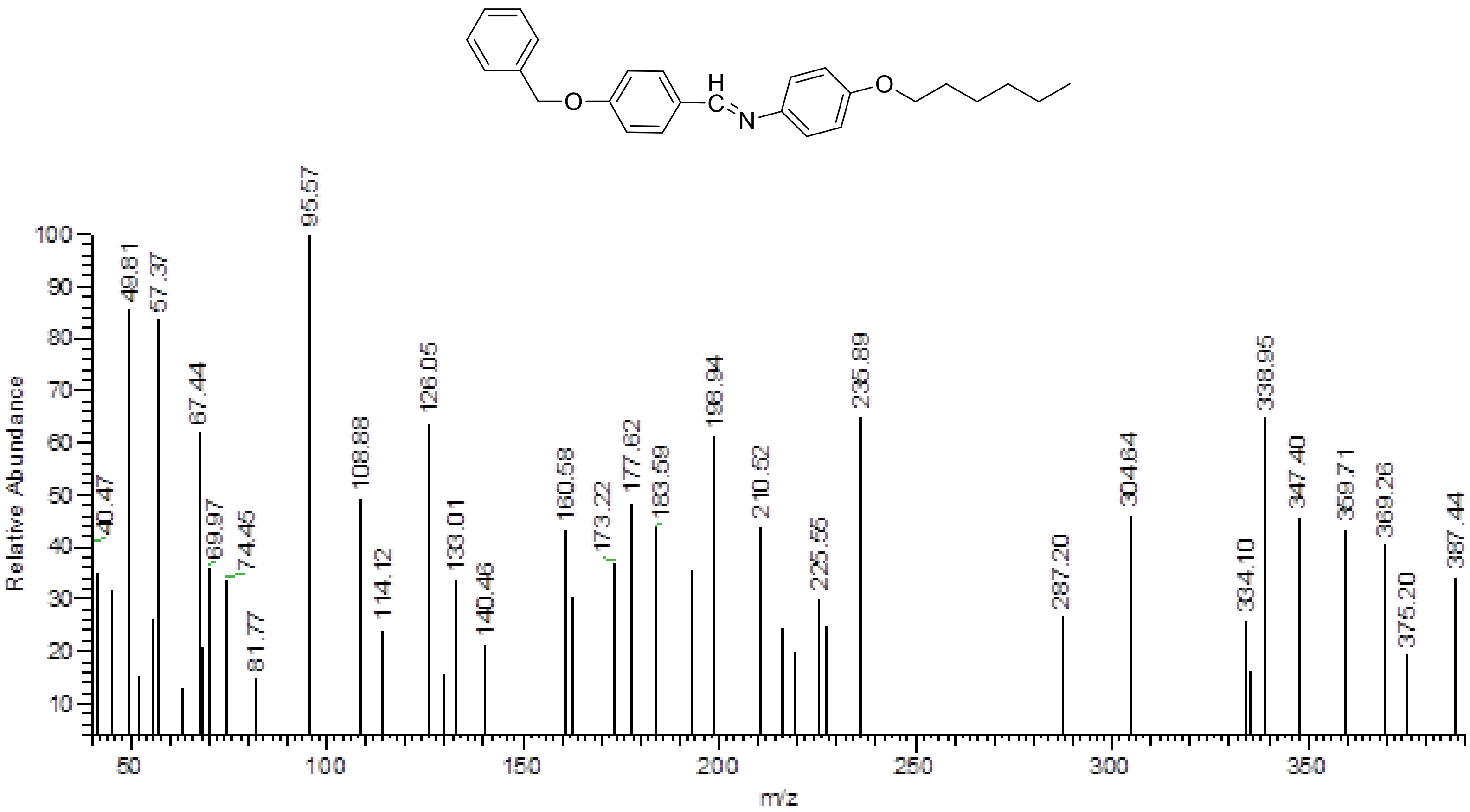

Figure S6. Mass spectrum of (*E*)-4-(hexyloxy)-*N*-(4-(benzyloxy)benzylidene)aniline $\mathbf{I_6}$

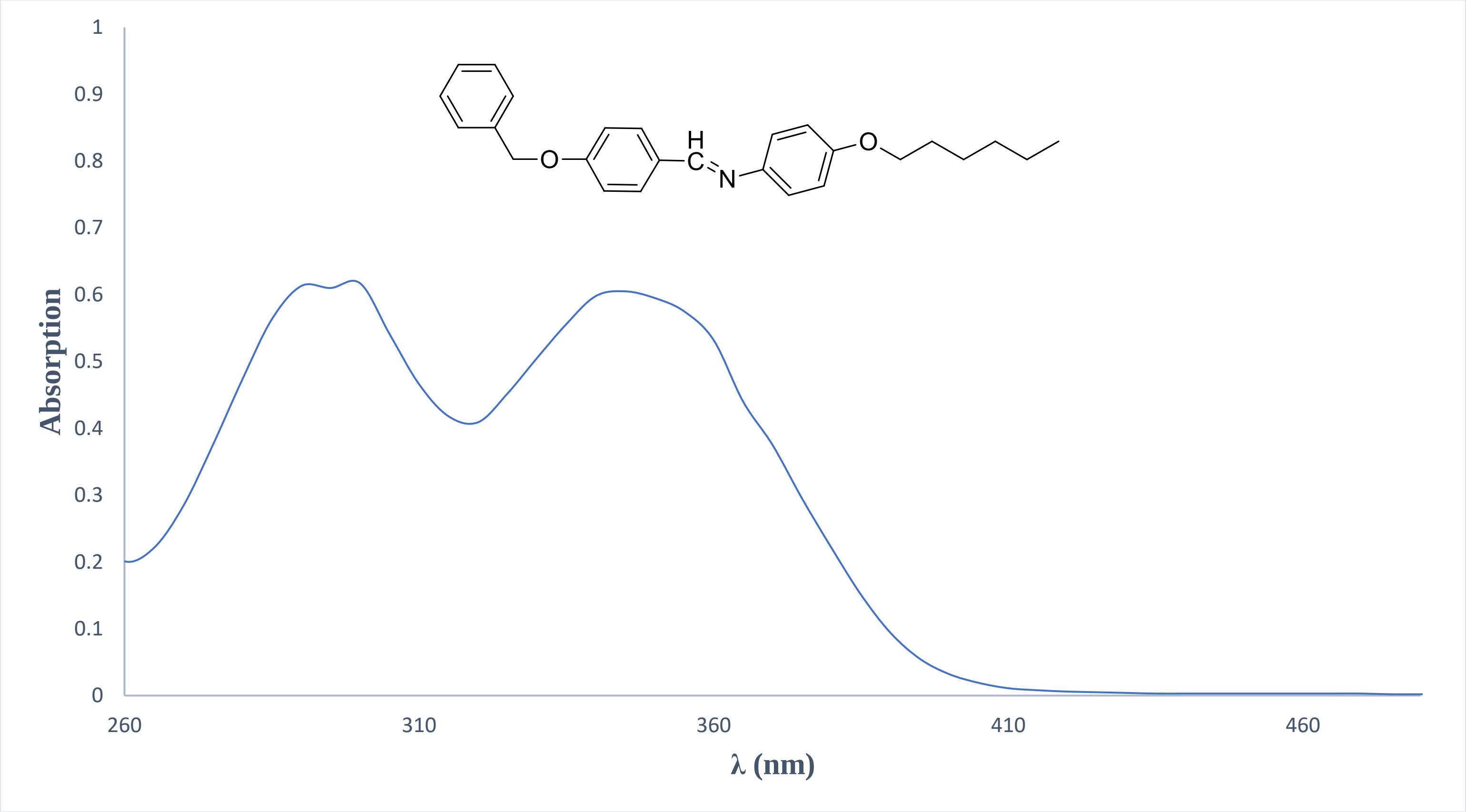

Figure S7. UV spectrum of (*E*)-4-(hexyloxy)-*N*-(4-(benzyloxy)benzylidene)aniline $\mathbf{I_6}$ in methanol

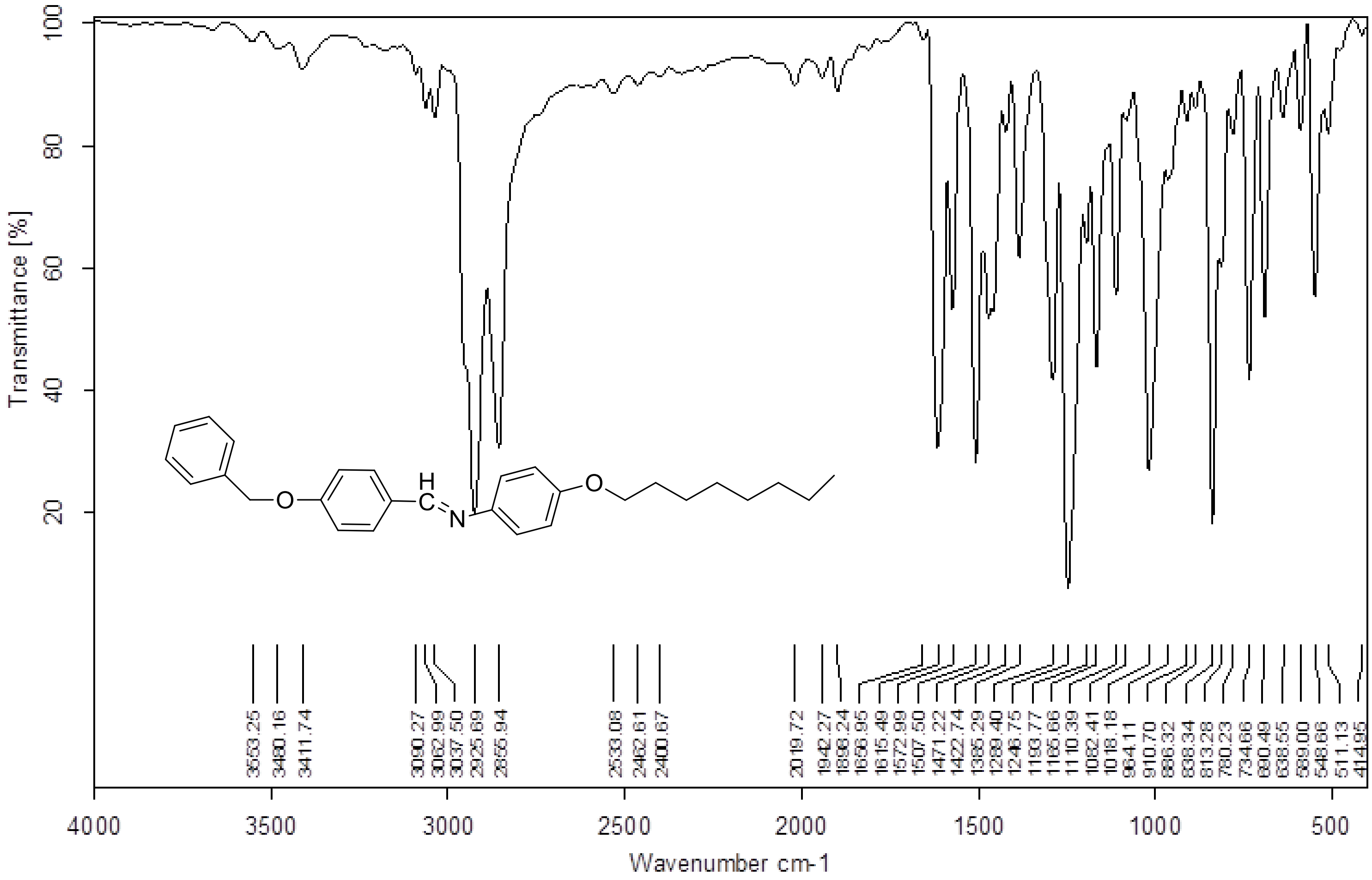

Figure S8. IR (KBr) spectrum of (*E*)-4-(octyloxy)-*N*-(4-(benzyloxy)benzylidene)aniline **I8**

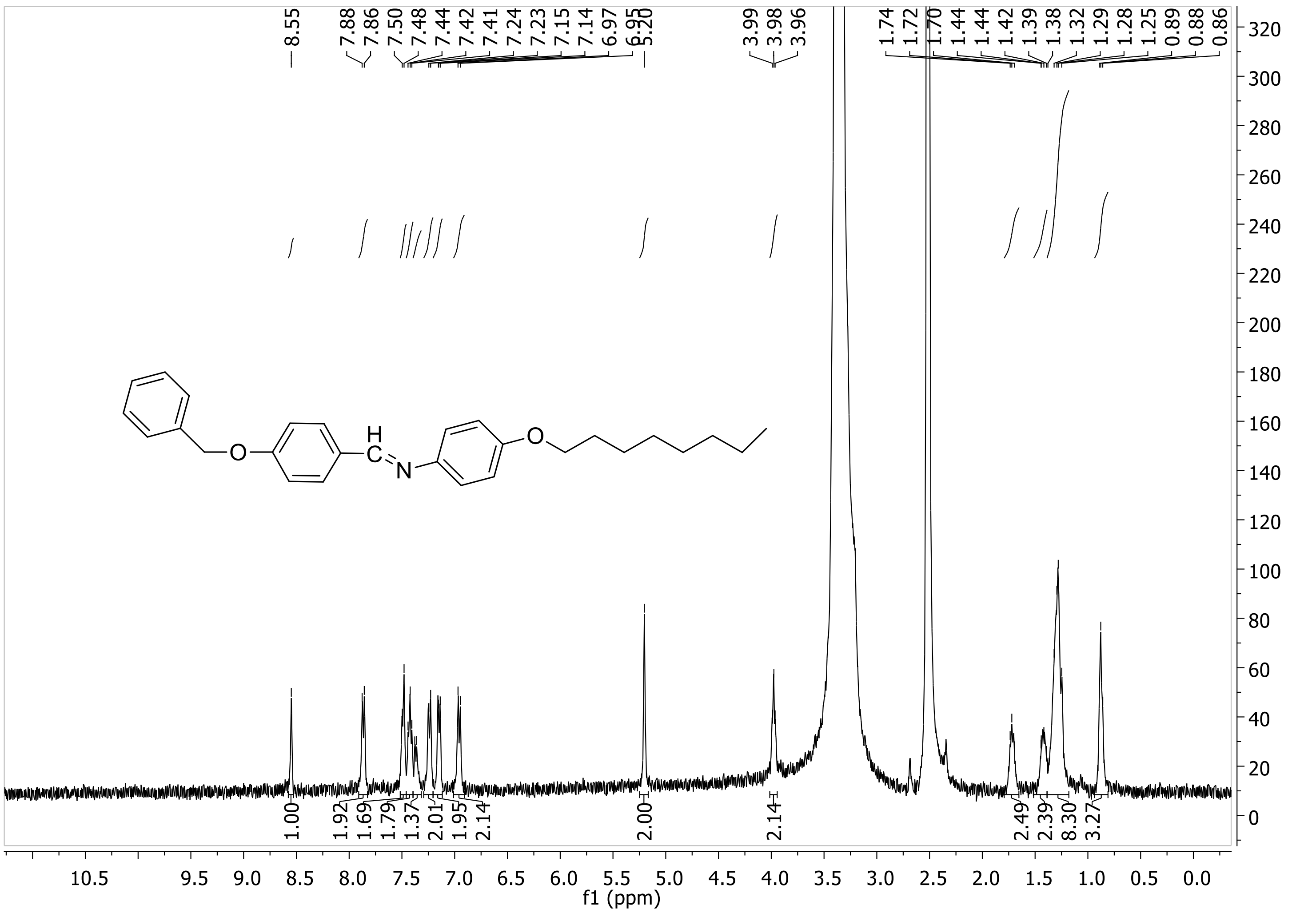

Figure S9. ^{1}H NMR (DMSO-*d6*) spectrum of (*E*)-4-(octyloxy)-*N*-(4-(benzyloxy)benzylidene)aniline **I$_8$**

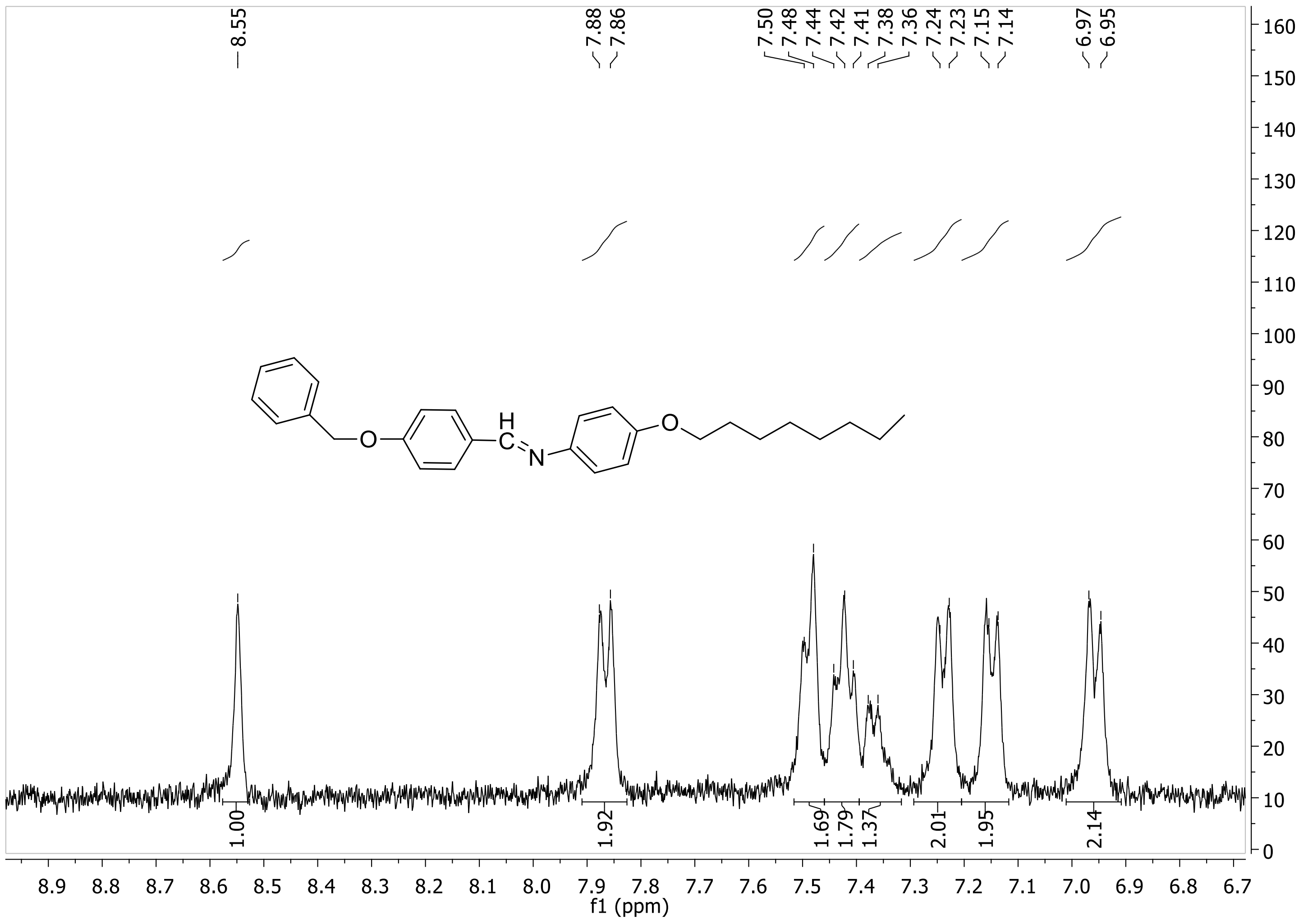

Figure S10. ^{1}H NMR (DMSO-*d6*) spectrum of (*E*)-4-(octyloxy)-*N*-(4-(benzyloxy)benzylidene)aniline **I₈**

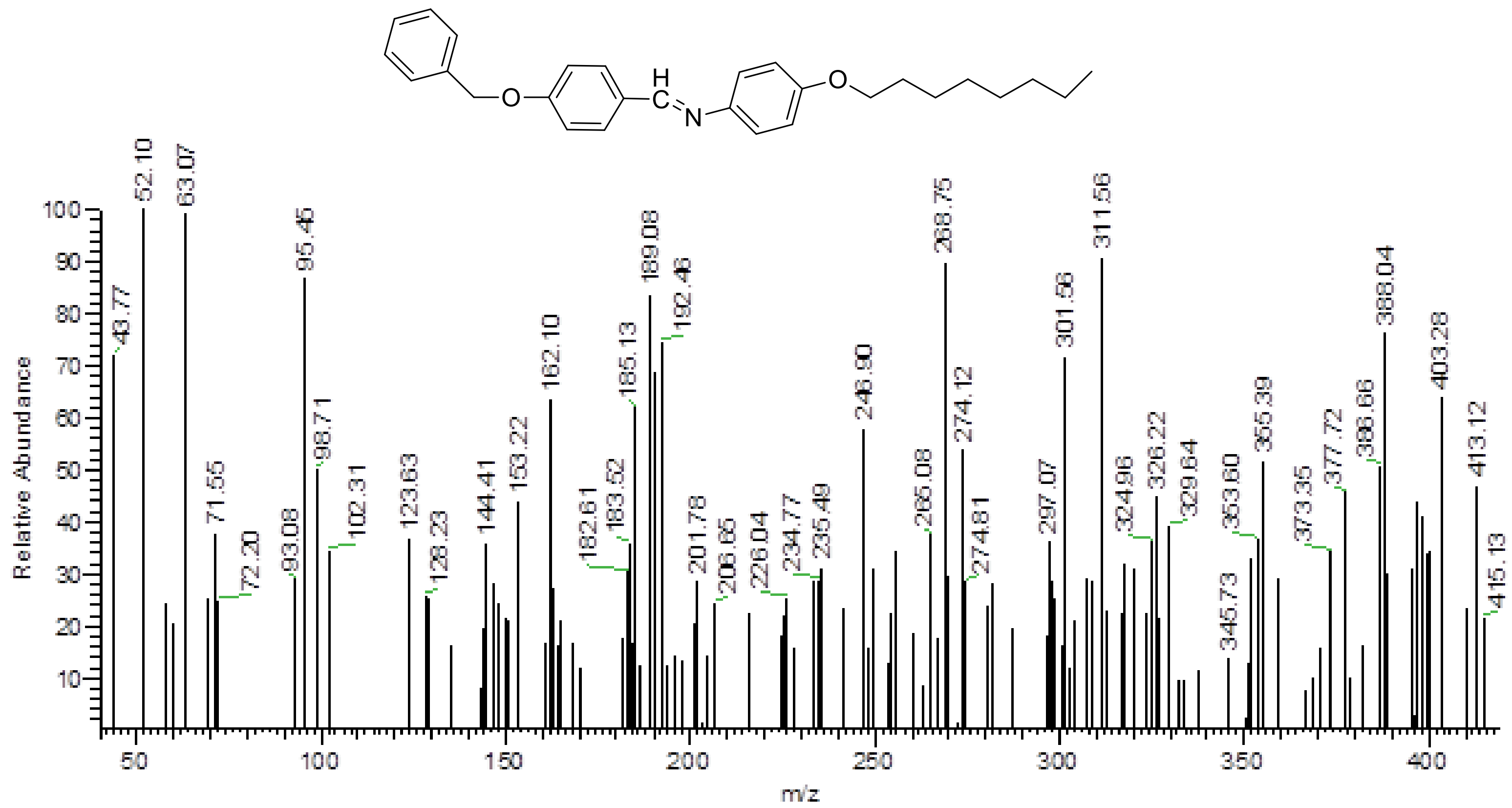

Figure S11. Mass spectrum of (*E*)-4-(octyloxy)-*N*-(4-(benzyloxy)benzylidene)aniline **I$_8$**

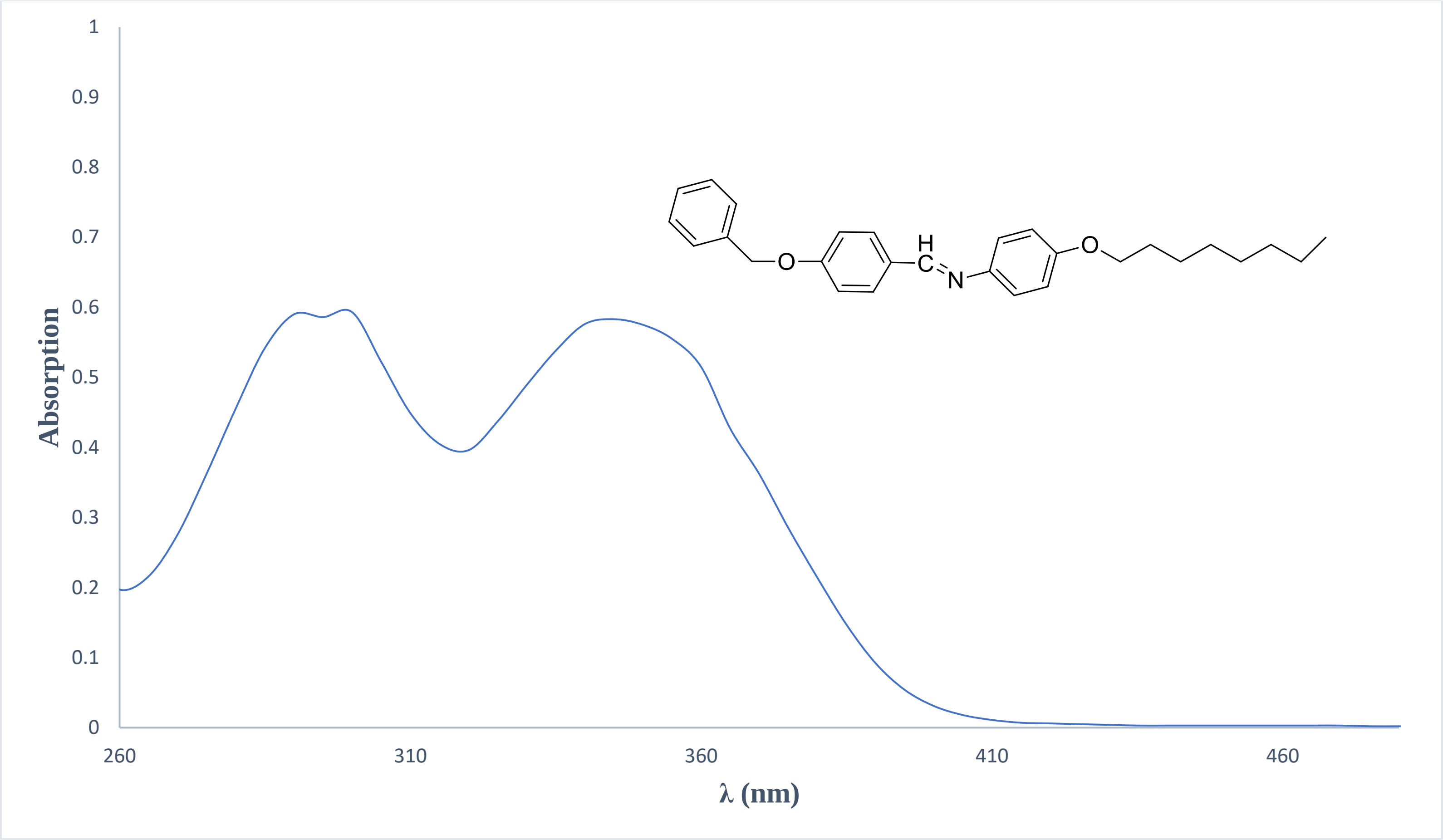

Figure S12. UV spectrum of (*E*)-4-(octyloxy)-*N*-(4-(benzyloxy)benzylidene)aniline $\mathbf{I_8}$

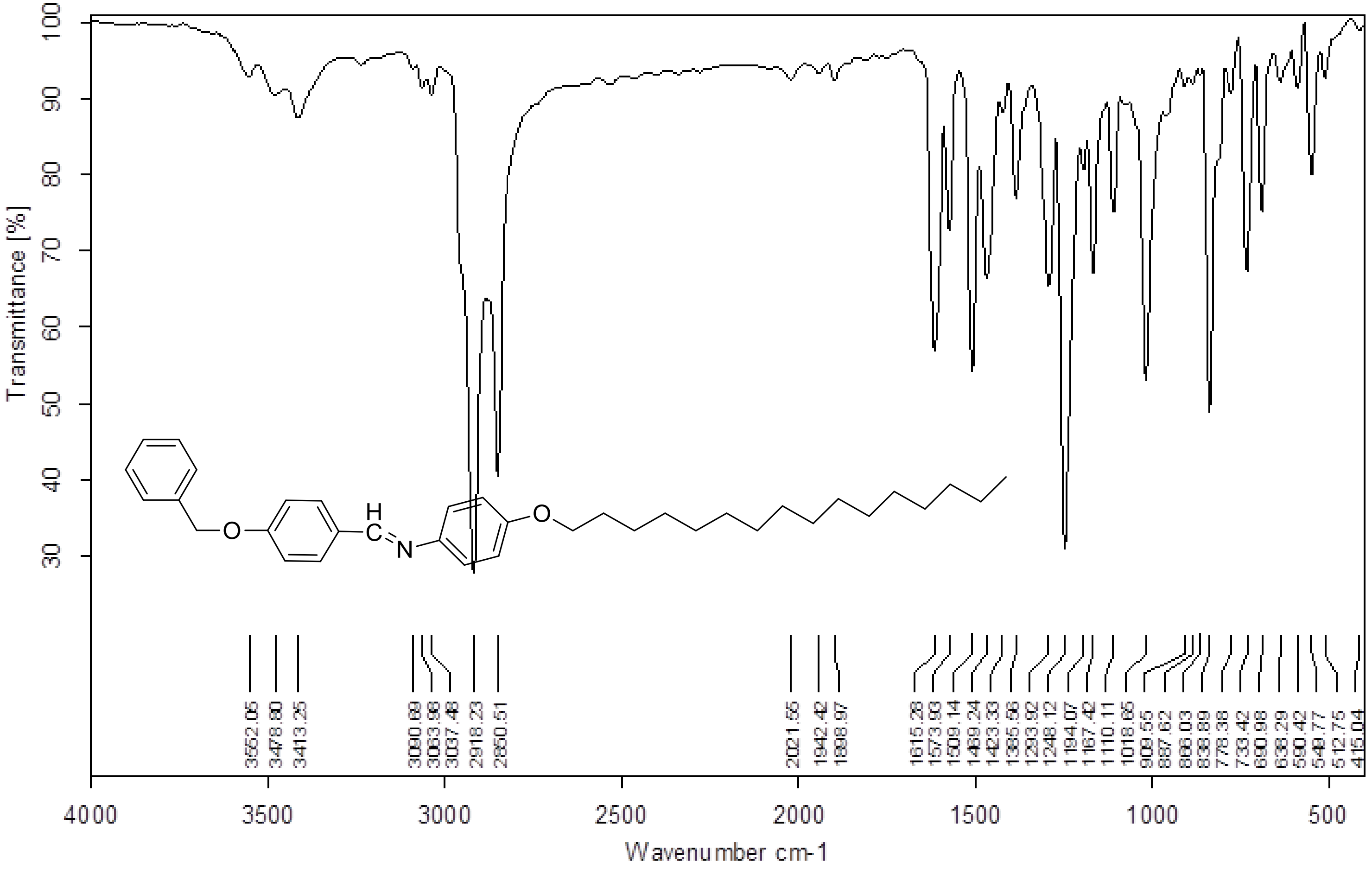

Figure S13. IR (KBr) spectrum of (*E*)-4-(hexadecyloxy)-*N*-(4-(benzyloxy)benzylidene)aniline **I$_{16}$**

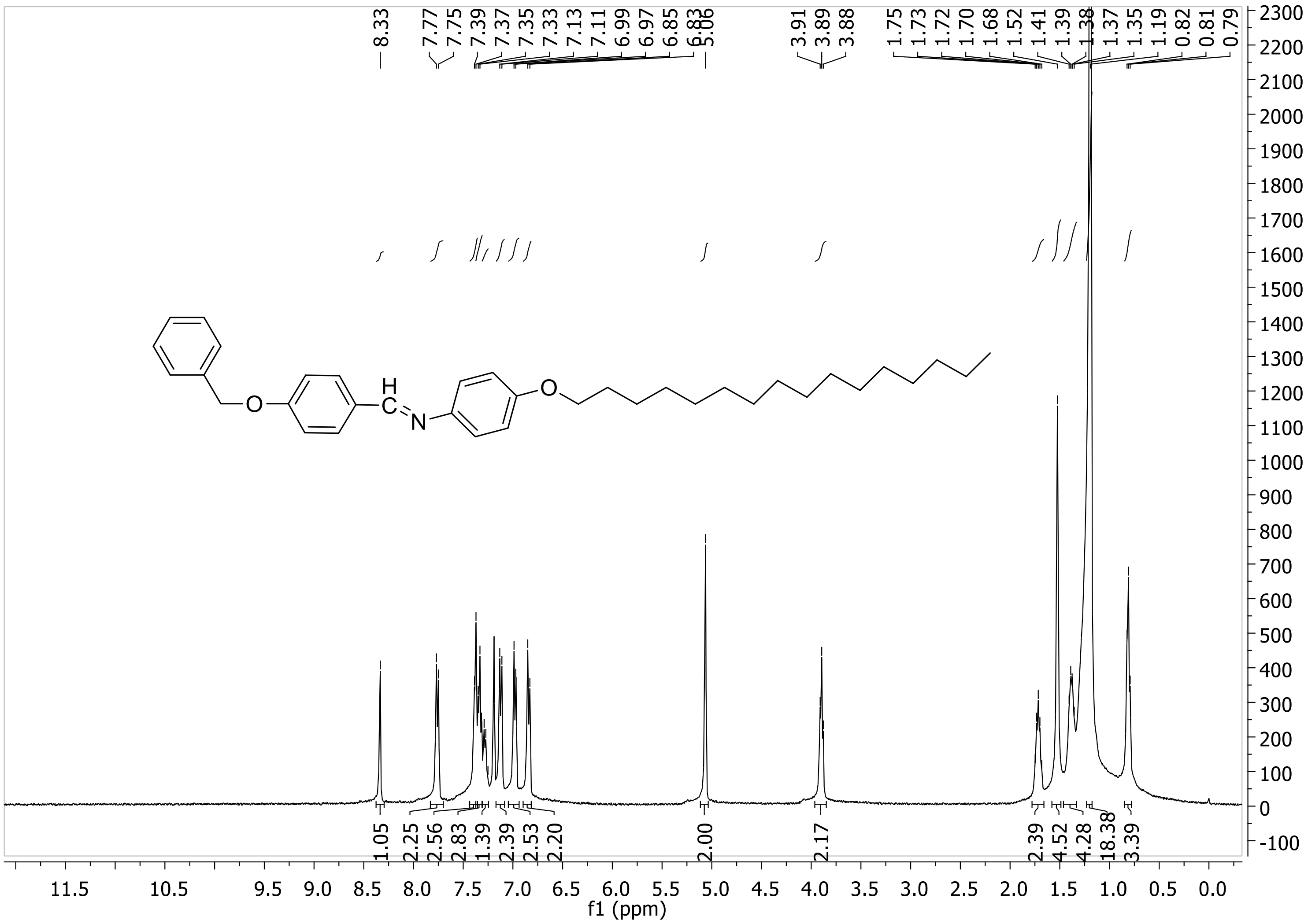

Figure S14. ^{1}H NMR ($CDCl_3$) spectrum of (*E*)-4-(hexadecyloxy)-*N*-(4-(benzyloxy)benzylidene)aniline **I$_{16}$**

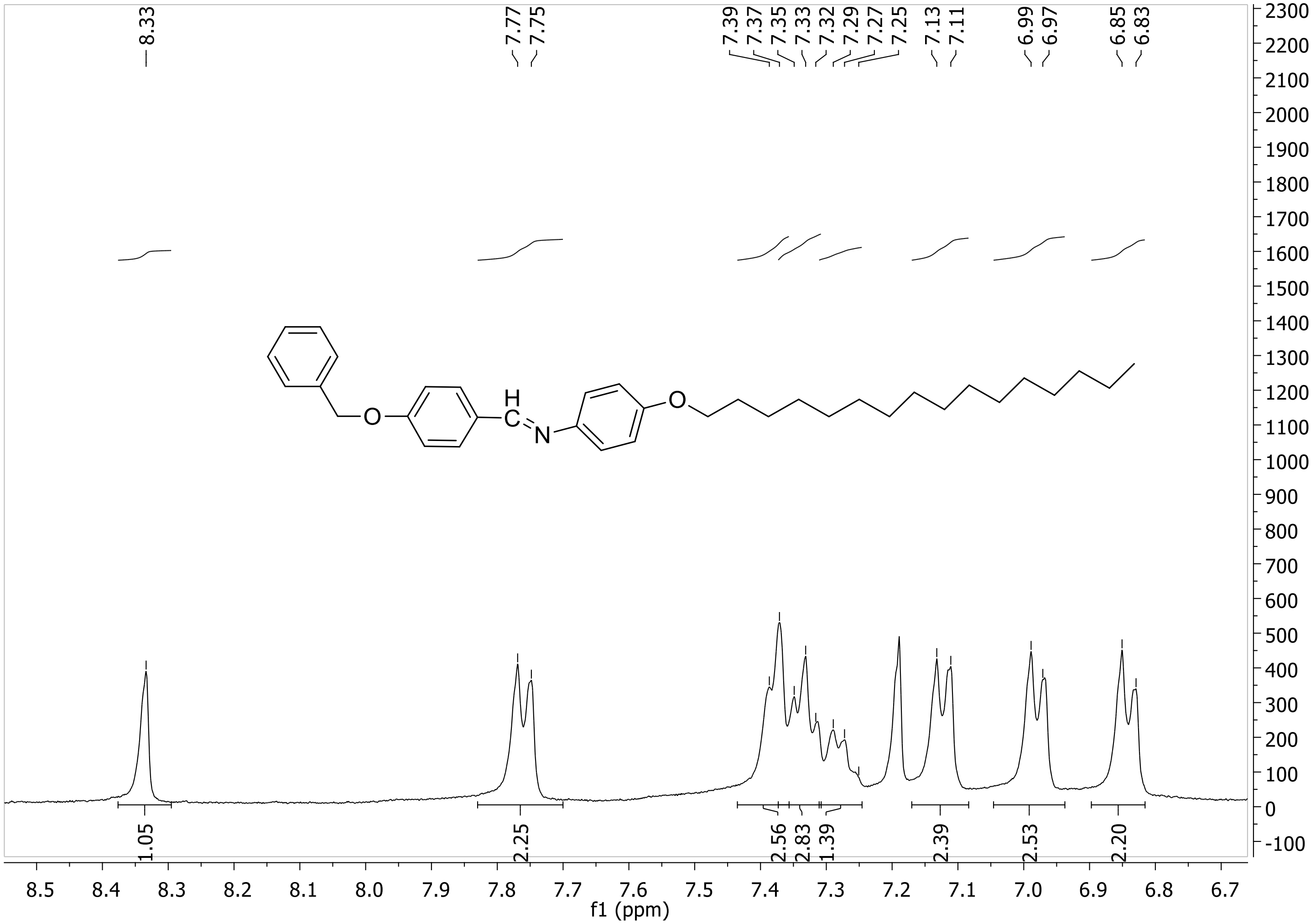

Figure S15. ^{1}H NMR ($CDCl_3$) spectrum of (*E*)-4-(hexadecyloxy)-*N*-(4-(benzyloxy)benzylidene)aniline **I_{16}**

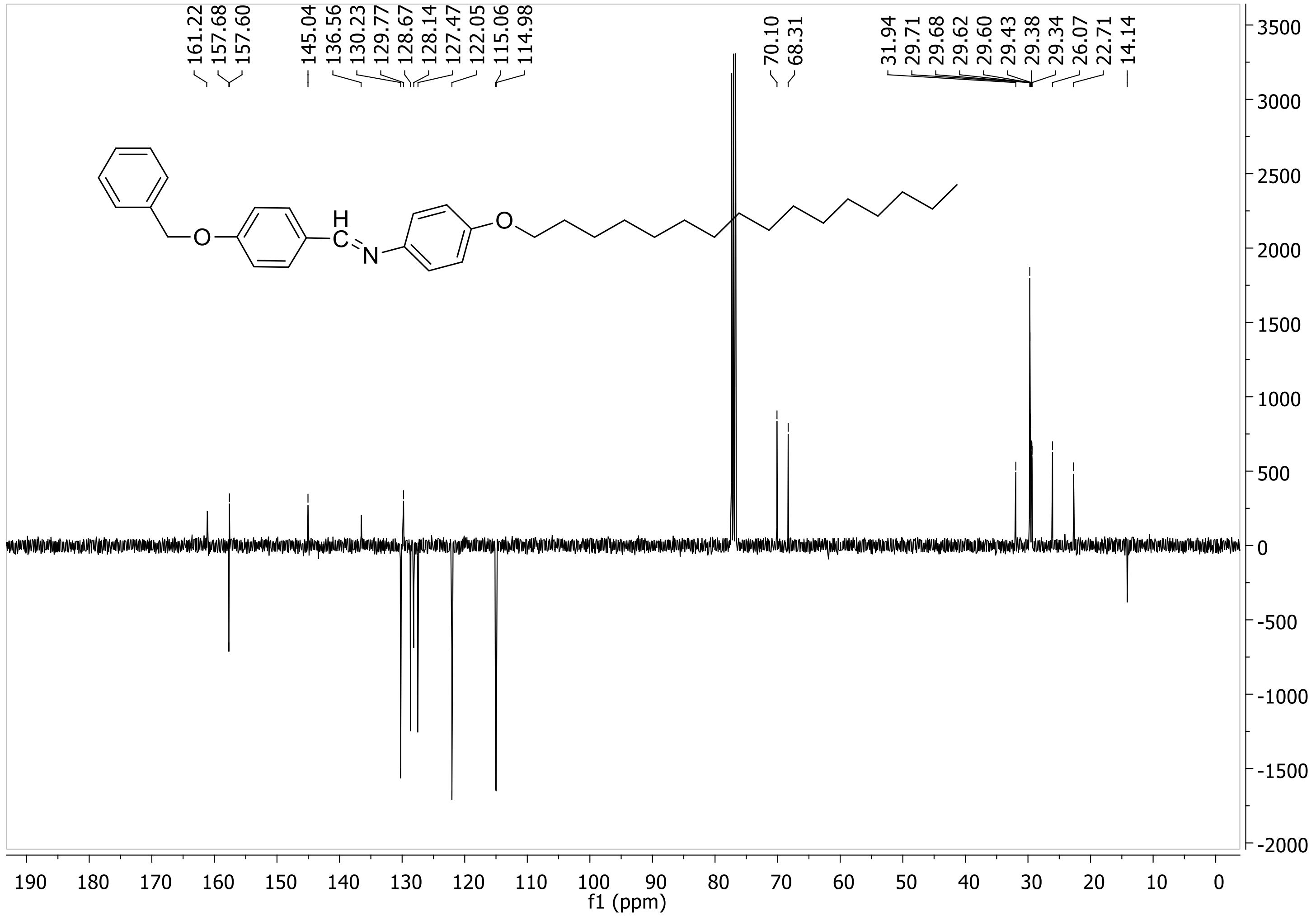

Figure S16. ^{13}C APT NMR ($CDCl_3$) of (*E*)-4-(hexadecyloxy)-*N*-(4-(benzyloxy)benzylidene)aniline **I$_{16}$**

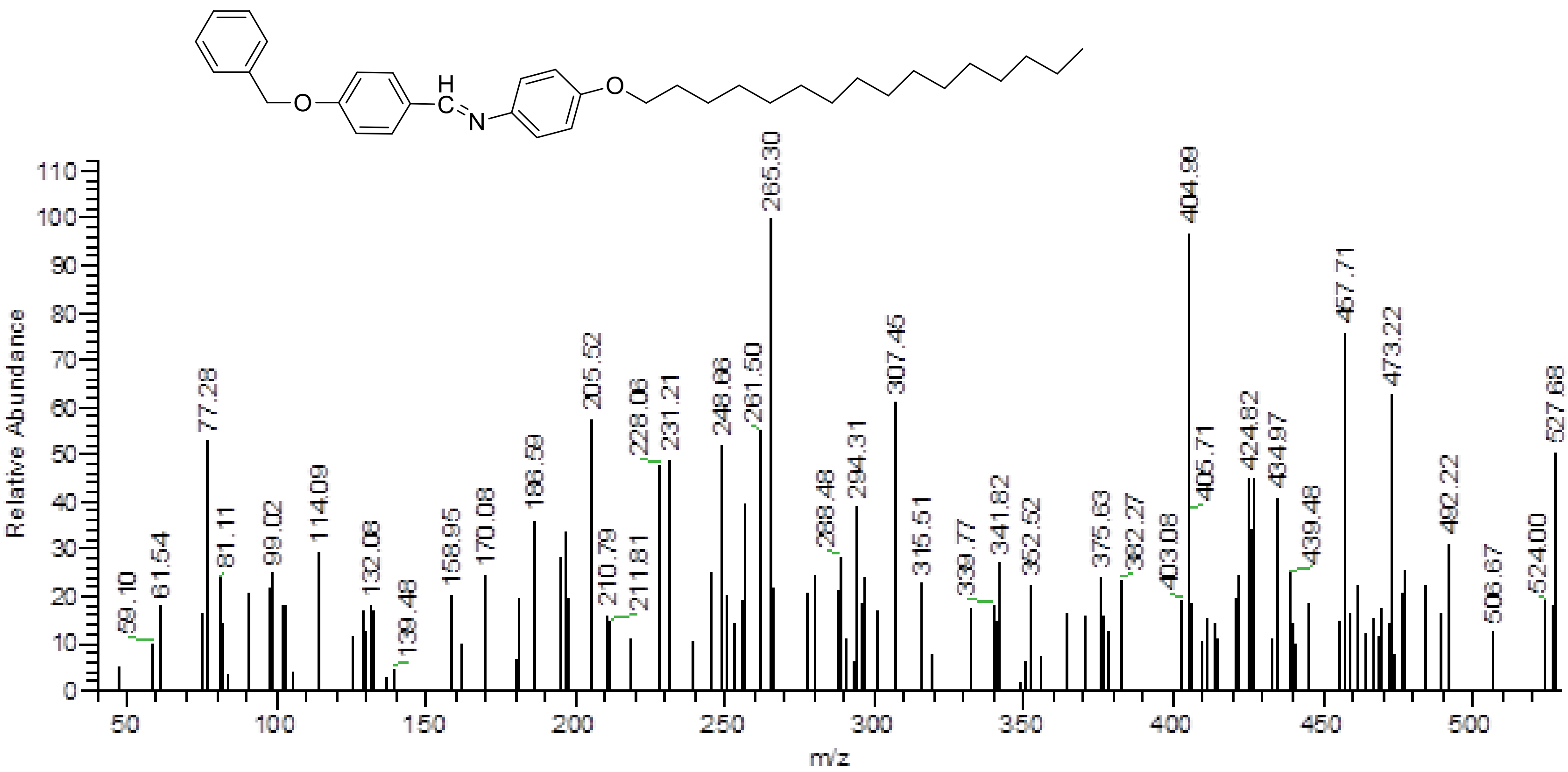

Figure S17. Mass spectrum of (*E*)-4-(hexadecyloxy)-*N*-(4-(benzyloxy)benzylidene)aniline **I$_{16}$**

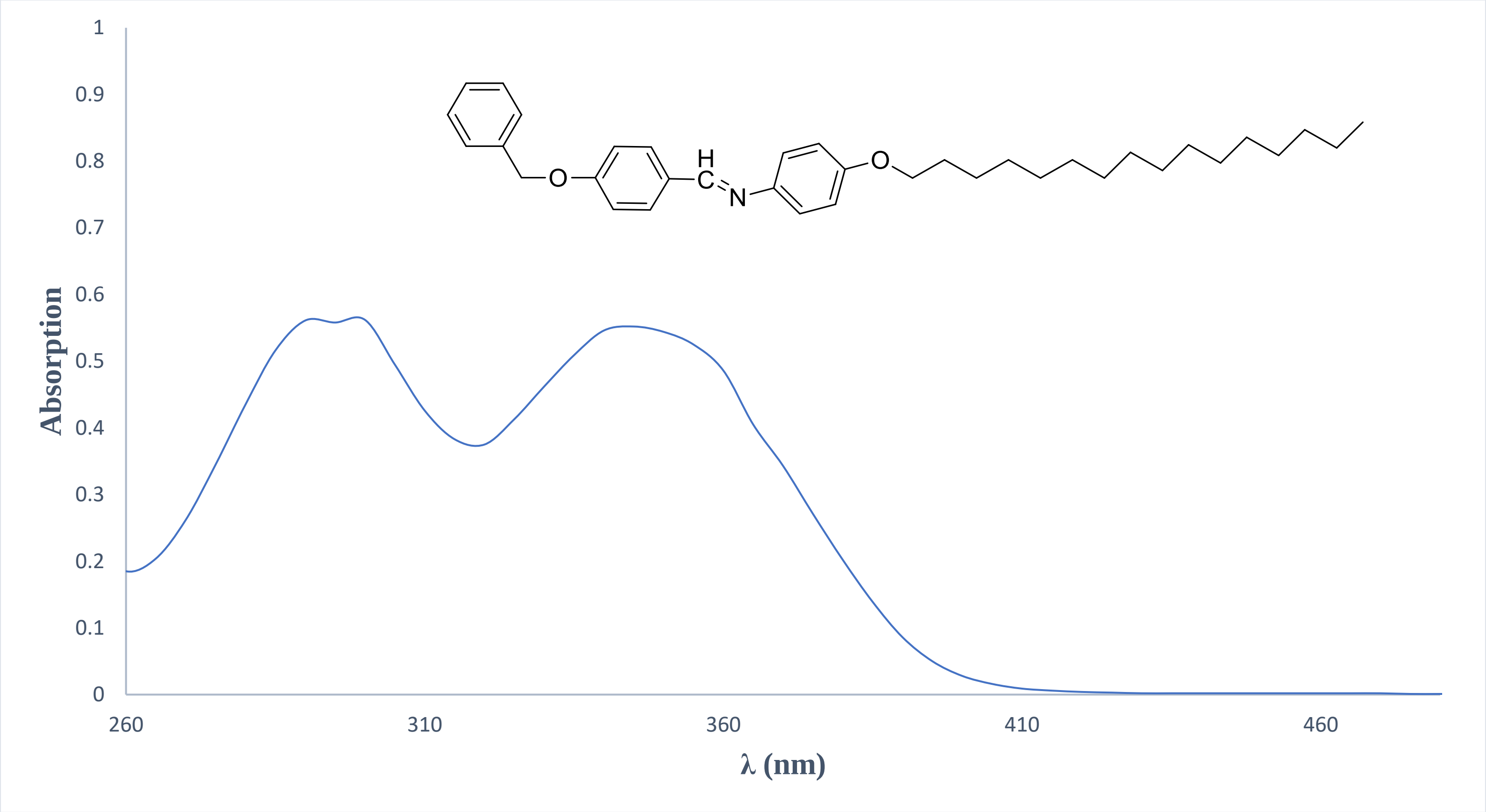

Figure S18. UV spectrum of (*E*)-4-(hexadecyloxy)-*N*-(4-(benzyloxy)benzylidene)aniline $\mathbf{I_{16}}$ in methanol
